# Effectiveness of an Academic-Practice Team Approach on Research Capacity Building of Nurses and Public Health Professionals

**DOI:** 10.3390/ijerph18137199

**Published:** 2021-07-05

**Authors:** Yuwadee Wittayapun, Jiraphat Nawarat

**Affiliations:** Department of Physical Therapy, School of Allied Health Sciences, Walailak University, Nakhon Si Thammarat 80160, Thailand; nsuparoe@wu.ac.th

**Keywords:** research capacity building, academic-practice partnerships, nursing program, health professional

## Abstract

The purpose of this study was to determine the need for research training among nurses and health professionals in a rural province of Thailand and to evaluate the effectiveness of the interventions designed to address the identified factors. This two-phase study used a cross-sectional design with one-group pre- and post-tests. In phase I, 149 subjects from 16 subdistrict health promoting hospitals and one district hospital were sampled. As an intervention, an academic-practice team approach to research capacity building was designed. Twenty-four volunteers completed a three-time point assessment of intervention in phase II. Data were collected using self-report questionnaires and analyzed using bivariate and multivariate statistics. Phase-I results indicated that 33.6% of subjects were involved in the research implementation. They had a moderate perception of research barriers and capacity. The research experiences, capacity, and barriers associated with the research implementation were described in detail (*p* < 0.05). The only positive predictor of research implementation was research training (*p* < 0.001). The intervention improved 24 participants’ competency (*p* < 0.05). Most of their research proposals had received ethics approval and a small grant. These findings highlight the efforts of innovative research capacity development and its impact on research and health practices among nurses and health professionals.

## 1. Introduction

High-quality research has been a boon to significantly improve the quality of care and achieve cost-effective health outcomes for patients, families, healthcare providers, and the healthcare system [1,2]. Nurses and various public health professionals are increasingly expected to make evidence from scientific studies available to evidence-based practice (EBP), but this expectation has not yet been fulfilled [3,4,5]. Barriers to the use of research findings in practices are the organizational context (e.g., insufficient time, low authority to change, inadequate facilities), and the presentation of evidence (e.g., statistics, compatibility to understand context, and availability of evidence), and characteristics of users (e.g., unaware of research, incapable of evaluating research, isolated from knowledgeable colleagues) [6,7]. Similarly, related studies identified barriers to research participation, such as time constraints [4,8], staff shortages [9] or overwork [8], lack of knowledge/skills [8,10], funding [4,8], and lack of supervision and research training [4,11].

Research capacity building (RCB) throughout health professionals’ careers is perceived as a priority and contributes to promoting research implementation, EBP, and strengthening health systems’ research [5,12]. RCB is a process of individual and institutional development aimed at increasing the capacity to conduct high quality research [13]. Implementing research and RCB has long been recognized as a critical step toward resolving inequalities and inequitable access to health services, but is frequently overlooked [14,15]. A systematic review of 42 health research capacity studies by Huber et al. [16] showed that the majority were either conducted on the individual/team or both individual/team and organizational level. However, 30 research studies were nonintervention. Additionally, in low- and middle-income countries, needs assessments are rarely reported (LMICs) [16]. A few pieces of available evidence have attempted to investigate needs assessments [4,17] or provide programs to strengthen the research capacity of nurses [18] and public health professionals [19], particularly those providing community and primary healthcare services [10,20].

Academic institutions have the potential to play a crucial role in breaking down these research barriers and facilitating research initiatives [21]. Academic inputs can contribute to many significant projects aimed at developing new models of care, improving access to services, supporting trained health professionals, or building capacity in organizations and communities [22]. In Thasala District, public health facilities under the supervision of the Ministry of Public Health include a community hospital (120 beds) and 16 subdistrict hospitals. Nurses and public health professionals work primarily in communities and primary care settings near a university, with little interest in research activities. With the ultimate goal of promoting EBP research, questions arise regarding what current research participation is and how to offer RCB to effectively use and generate useful evidence that could have a greater impact in this setting.

Various strategies have been proposed to support RCB and reduce barriers, including strengthening individual research skills [20,23], using a team-based approach [13,24], supporting research-based health practices [23], receiving organizational support [13], providing a small grant [20], assisting with research mentoring [20], writing for publication [20], and developing international networks [25]. A critical recommendation of research capacity development is to collect contextually relevant data for planning, assessing, monitoring, and evaluating processes [5,16]. As such, an innovative RCB intervention should be designed according to identified essences and reviewed literature. Hence, the objectives of this two-phase study were to determine the nursing and health professional needs for research training, implementation, barriers, and capacity. Additionally, this study sought to specifically assess the effectiveness of the innovative RCB program, an academic-practice team approach, tailored to identify determinants in terms of changes in research-related competency, proposal writing, ethics approval, and finally dissemination.

This two-phase study begins with the findings of a needs analysis and then assesses the effectiveness of an academic-practice team approach on RCB. This could establish crucial information that is beneficial for future systematic improvement of the holistic approach to effective research development among health practitioners in other settings to promote the quality of healthcare delivery.

## 2. Methods

### 2.1. Study Design

This two-phase research project included a cross-sectional study conducted from October to December 2013 and a second part with a one-group pretest-posttest design conducted from December 2013 to September 2014. The overall study was conducted at Walailak University in Tha Sala District, in Nakhon Si Thammarat Province of southern Thailand.

### 2.2. Participants and Sample Size Estimation

#### 2.2.1. Phase I

**Figure 1:**
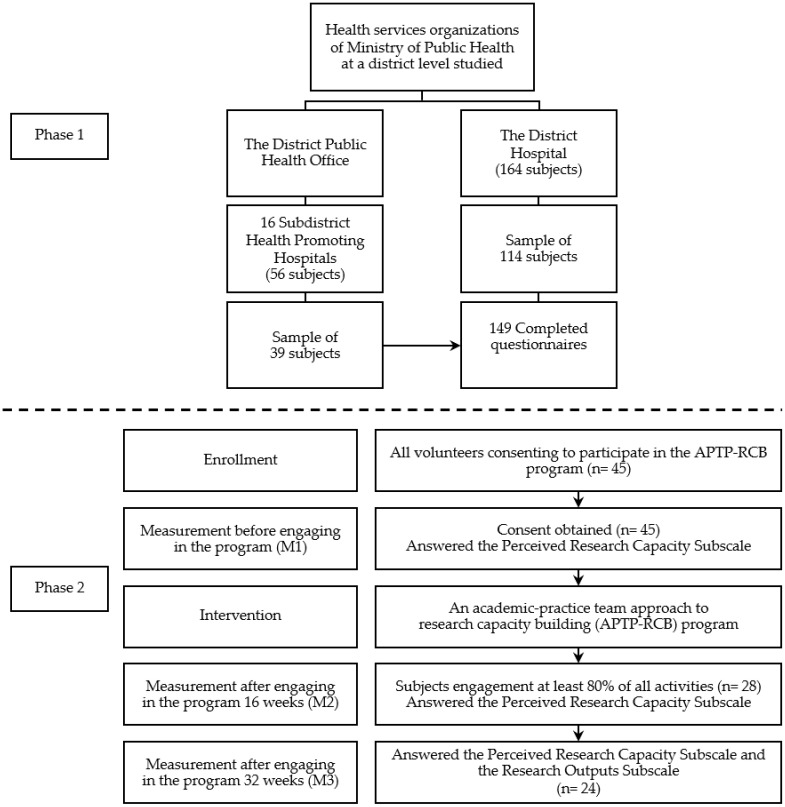
Flow diagram of participants and data collection in phase 1 and 2 studies.

The study population included nurses and other health professionals employed by the Ministry of Public Health in Thasala District, Nakhon Si Thammarat Province, Thailand, where Walailak University is located. At the district level, the health service organizations consists of two departments: the district hospital and the District Public Health Office. The latter comprises 16 subdistrict health promoting hospitals. Several criteria were used to determine inclusion: (1) Registered nurses, public health technical officers, public health officers, medical technologists, medical science technicians, physiotherapists, and occupational therapists were all included in the study/healthy population, and (2) between the ages of 22 and 60. A total of 220 subjects were recruited; 56 worked at 16 subdistrict health promotion hospitals, and 164 worked at the district hospital. The sample size was calculated using a formula that estimates the proportion of a finite population [26], and based on the results of a pilot study, whereas the proportion of the conducting research was 26%, the confidence level was set at 95%, and the estimated error was 5%. The calculated sample size was 127. Recognizing incomplete questionnaires returned [10], we opted for 153 (Figure 1). With the collaboration of each department, a list of all research populations was created. This was used for a proportional stratified random sampling technique. Though a cross-sectional study cannot identify a causal inference in a snapshot situation, the design helped us understand factors related to research implementation being beneficial to tailor an innovative intervention in Phase II [27].

#### 2.2.2. Phase II

A quasi-experimental study was used to assess participants’ research capacity. Though randomized controlled trials would be desirable for higher internal validity, randomly allocating participants to intervention or control groups is not easy, particularly in limited health resources settings. Thus, this study adopted a single group of pre- and posttest designs to assess the extent to which an intervention can be effectively integrated within real-life systems [28].

Twenty-four subjects were chosen for the study based on the desired power level of 0.80, the effect size of 0.7, and the alpha level of 0.05 [29]. The inclusion criteria for participants included nurses and health professionals who applied voluntarily to be a part of the program, had at least two years’ experience working in public healthcare facilities located in this province, and received approval from their organizations before the study’s start. Recognizing the time-consuming intervention and systematic variation sample attrition [2], a total of 45 subjects who were interested in and consented to participate in the projects in the first round of the program were welcomed. Finally, a total of 24 subjects did not violate the exclusion criteria, requiring that at least 80% of all activities be performed, and completed all questionnaires (Figure 1).

### 2.3. Measurement and Data Collection

#### 2.3.1. Phase I

The research instruments were guided by the BARRIERS scale [6], a framework for evaluating research capacity building [5], and publicly available literature [30], and was designed as a self-reporting scale including four subscales.

The personal factors subscale (PFS) consisted of six structured questions about age, education level, marital status, monthly income, work department, and work position. The research experiences subscale (RES) consisted of six questions regarding prior training in research-related fields, attendance at research conferences, application of research findings in practice, ongoing projects, project implementation, and interest in future training. The perceived research capacity subscale (PRC) consisted of 30 items (q1–q30) and was divided in 5 sections: (1) research problems, purpose, and hypotheses (RC1-RpPH, five items), (2) literature reviews and research framework (RC2-LrRf, six items), (3) research method (RC3-Rm, eight items), (4) data collection, analysis, and interpretation (RC4-DcAI, four items), and (5) findings, discussion and dissemination (RC5-FdD, 7 items). The perceived research barriers subscale (PRB) consisted of 24 items (b1–b24), including both personal (RB1-Pb) and organizational (RB2-Ob) barriers (12 items, equally). PRC and PRB were designed with responses on five-point Likert scales. Three experts evaluated the content validity: two professionals (lecturers at Mahidol University, Salaya, Thailand) and a lay expert (a nurse working in the hospital). PRC and PRB had Cronbach’s alpha reliability coefficients of 0.94 and 0.93, respectively.

#### 2.3.2. Phase II

An academic-practice team approach to the research capacity building (APTP-RCB) program was developed according to the critical gaps in perceived competence and identified barriers and from the related literature [5,13,20] and focused on the academic-practice research team approach. The program comprised five strategies. A nondegree training intervention was initially offered to help participants develop the necessary research capacity for 32-week commitments. The design highlighted appropriate contents and research methods and focused on discovering competency weak points including research related to writing a manuscript, preparing instruments, selecting appropriate statistics, project designing, interpreting findings, and conceptualizing a research framework. Therefore, a research training course was held in eight sessions of two-day classes scheduled monthly. Second, a small grant was allocated from the APTP-RCB program to write a small-scale research project, constantly depending on its research design. Survey research projects received 8000 THB (About 256 USD) and quasi-experimental research of 10,000 THB for grants. Third, nurses and public health professionals working in public health facilities located in Nakhon Si Thammarat Province expressed their engagement as members of an academic-practice research team and described the research idea close to health-related problems encountered. Eligible participants were required to provide their leaders/superiors’ approval to participate in the APTP-RCB intervention. Fourth, healthcare organizations collaborated by providing free time for their staff to participate in the program and conduct the research. Last, health faculty members from Walailak University and the network (i.e., Mahidol University and Boromrajonani College of Nursing) were coordinated and invited to be a part of research teams and serve as academic mentors for potential research projects. Meetings between mentors and mentees were arranged throughout the project, taking place face-to-face, by email, or telephone. While participants had to report their research progress, mentors had to monitor and facilitate the research team to complete research proposals, submit them to the ethics committee, and write research reports/manuscripts. Accordingly, invitation letters were sent to the provincial public health office and provincial hospital to inform the objectives of the APTP-RCB, invite public health personnel to volunteer for the program, and ask for the organization’s collaboration. To assess the effectiveness of the APTP-RCB program in perceived research competence, the PRC was used to collect data at three time points of engagement in the program: before (M1), after 16 Weeks (M2), and after 32 Weeks (M3). Additionally, research outputs were followed up using a research outputs subscale (ROS) at the end of the program. The ROS embraced five-item structured questions to obtain research approval from the institutional review board, receiving research related to a small grant, design, targeted sample, and dissemination.

### 2.4. Statistical Analysis

All data were analyzed using the statistical package software SPSS (Version 23, Chicago, IL, USA). Descriptive statistics, including frequency, percentage, standard deviation, skewness, and kurtosis, was performed to examine the accuracy of data entry, assess basic assumptions, and present general information about the sample and studied variables. Assumptions of the logistic regression analysis were tested [31]. According to the PRC and PRB subscale, the rating scores of 5, 4, 3, 2, and 1 were given to each item response. To aid in interpreting data, the mean of each item was classified in four categories: 1.00–1.49 was the lowest level, 1.50–2.49 was a low level, 2.50–3.49 was a moderate level, and 3.50–4.00 was the highest level. The mean score for the subscale’s overall items and each dimension was estimated to be 100. This was classified in four categories: less than 25.0 represented the lowest level, 25.0–50.0 represented a low level, 50.1–75.0 represented a moderate level, and 75.1–100 represented the highest level. The relationship or difference between personal characteristics, research experience, and research implementation, was determined using the *t*-test, Mann–Whitney test, or Chi-square test, as applicable. A logistic regression analysis was used to determine the factors influencing the implementation of the research. The Friedman and Wilcoxon signal rank tests were used to assess the effect of the APTP-RCB before, and after 16 and 32 weeks of APTP-RCB interventions, respectively. The level of significance was set at *p* < 0.05.

## 3. Results

### 3.1. Phase I of the Study

We analyzed a total of 149 completed questionnaires. The average age of respondents was 37.6 years (±9.8); the majority of participants were female (85.2%), had a bachelor’s degree or less (91.9%), were married/separated/divorced (63.1%), and earned more than 30,000 THB monthly (55.7%). The average number of years worked by respondents was 14.9 years (±10.1); the majority (71.8%) worked at the district hospital and were professional nurses (64.4%) (Table 1).

#### 3.1.1. Research Experiences and Research Implementation

Nearly one half had been trained in research (45%). Two thirds of subjects (67.8%) had attended research conferences and applied the findings (59.1%). One third of subjects had already implemented a research project (33.6%), including having a project undertaken (6.7%), and two thirds of respondents were interested in future research training (69.1%) (Table 1).

#### 3.1.2. Perceived Research Capacity (PRC) and Perceived Research Barriers (PRB)

Overall, the participants had a moderate level of PRC (mean 58.2 ± 12.8). The RC2-LrRf had the highest mean (mean 60.2 ± 13.7), while the RC5-FdD had the lowest mean (mean 55.9 ± 14.3). Each PRC item had a mean in the moderate range (2.6–3.2). One item, writing a research manuscript, was the lowest among the five items holding the lowest mean. The overall PRB was at a moderate level (64.2 ± 11.2). The RB2-Ob had a higher mean than RB1-Pb (66.6 ± 12.8 and 61.8 ± 12.0, respectively). The mean range of PRB items was 2.3–3.9. Reading research literature in English was ranked first among the top five barriers to RB1-Pb. Additionally, workload constraints were the first of RB2-Ob’s top five barriers (Table 2).

#### 3.1.3. Relationship between Personal Factors, Research Experiences, and Implementation of Research

Personal factors did not show a statistically significant relationship with research implementation (*p* > 0.05). Research training, participation in conferences, application of findings, and interest in future research training had a statistically significant relationship with research implementation (*p* < 0.05). Participants who implemented research were trained, attended conferences, applied the research findings, and expressed an interest in future research training at a higher proportion than those who did not (Table 3).

#### 3.1.4. Relationship between PRC and Research Implementation

The overall PRC did not differ significantly between those who implemented the research and those who did not (Z = −1.158, *p* = 0.124). Regarding the PRC dimension, the RC1-RpPH and RC2-LrRf were statistically significant (Z = −1.676, *p* = 0.047 and Z = −1.762, *p* = 0.039, respectively), but the RC3-Rm, RC4-DcAI, and RC5-FdD were not. Participants who implemented research had a higher mean ranking of the RC1-RpPH and RC2-LrRf than those who did not (Table 4).

#### 3.1.5. Relationship between PRB and Research Implementation

The PRB, RB1-Pb, and RB2-Ob all demonstrated a significant correlation with research implementation (t = 2.125, *p* = 0.018, t = 2.297, *p* = 0.012; and t = −1.751, *p* = 0.042, respectively). The participants, who implemented the research, had a lower mean score of PRB, RB1-Pb, and RB2-Ob than those who did not (Table 5).

#### 3.1.6. Factors Affecting Research Implementation

After examining the multicollinearity of the independent variables, seven independent variables (i.e., training in research, attending conferences, use of the findings, RC1-RpPH, RB1-Pb, RB2-Ob, and interest in participating in future research training) were analyzed using logistic regression analysis. The result showed that research training was the only positively significant predictor of research implementation. Participants who received research training had a 5.8 times greater chance of implementing the research than those who did not (odds ratio, 5.8; *p* < 0.001) (Table 6).

### 3.2. Phase 2 of the Study

The average age of the 24 participants was 47.8 years (±4.3) and all of them were female. The majority of participants held a master’s degree (54.2%), and were married (79.2%), Almost half of participants earned more than 50,000 THB monthly (41.7%). The average number of years worked by respondents was 25.6 years (±4.9); the majority (58.3%) worked in a provincial hospital and were professional nurses (75.0%) (Table 7).

#### 3.2.1. Perceived Research Capacity (PRC)

Before engaging in the APTP-RCB intervention, the participants had a moderate level of PRC (mean 50.4 ± 15.7). The RC4-DcAI had the highest mean (mean 56.9 ± 16.4), while the RC5-FdD had the lowest mean (mean 49.8 ± 16.1). Each PRC item had a mean range between 2.3–3.3. The item of conceptualizing a research framework was the lowest among the five items, holding the lowest mean. (Table 8).

#### 3.2.2. PRC before and after 16 and 32 Weeks of Participation in the APTP-RCB

When mean scores for each domain (RC1-RpPH, RC2-LrRf, RC3-Rm, RC4-DcAI, and RC5-FdD) and PRC were compared across different time points during the APTP-RCB [i.e., before (M1), after 16 weeks (M2), and after 32 weeks (M3)], all mean scores saw statistically significant differences between M1, M2, and M3. When comparing M1 and M2, all mean scores at M2 were significantly higher than those at M1. For M2 and M3, only the mean scores of RC3-Rm, RC5-FdD, and PRC of M3 were significantly higher than those of M2. (Table 9).

#### 3.2.3. Follow-Up Outputs of APTP-RCB Intervention at 32 Weeks

After having participated in the APTP-RCB intervention for 32 weeks, participants formulated 24 research teams and revealed that 95.8% of research proposals were approved by the institutional review board at Walailak University, Boromrajonani College of Nursing or Maharaj Nakhon Si Thammarat Hospital, Nakhon Si Thammarat Province. The majority of research projects received a small grant (91.7%), were designed as quasi-experimental research (54.2%), and had health recipients as the targeted sample (66.7%). A few had presented research results in an academic conference (12.5%) (Table 10).

## 4. Discussion

### 4.1. Phase I

Building research capacity has been focused on as a critical component to promote EBP for nursing and health professionals [32,33]. However, research development is lacking among staff nurses and health professionals, particularly in limited-resource settings [17,34,35]. Our findings showed that 33.6% of participants had implemented their research projects, and only 6.7% had research projects being undertaken, which is consistent with related research, which found that 25% of community health staff experienced assisting research projects [10], and 15% of primary healthcare professionals had an active research project [14]. Generally, health professionals are required to conduct research for their career advancement [9,10]. Finch et al.’s study revealed that job classification level was a predictor of research engagement [36]. Congruence between professional and organizational research cultures appeared to affect career success [24]. Hence, a further inquiry topic was whether conducting related research contributes to respondents’ appraisals or not.

A positive characteristic of respondents who conducted research (i.e., they had been trained, attended conferences, and had used findings) was discovered in this study, similar to that conducted by Glynn et al., i.e., that previous research training and involvement in research (e.g., presenting a conference research paper, and having a peer-reviewed research publication) was associated with a more positive attitude towards primary care research and development [4]. Two domains of respondent’s competencies, including research problems, purpose, and hypotheses, as well as writing literature reviews and research framework appeared to be useful for research development, unlike Johnson et al., who identified limited skills across the research spectrum of health professionals [8]. Subjects of this study had overall PRC at a moderate level. These results confirmed that nurses and health professionals need to be involved in research. The possible reason was that the majority of the participants (91.9%) graduated with a bachelor’s degree or less and primarily had skills-based rather than research-based qualifications [10]. The disadvantages of their competencies were comparatively seen in related studies regarding creating a research proposal, designing questionnaires, analyzing qualitative and quantitative research data, and writing for publication in peer-reviewed journal analyses [8,10]. Two thirds of participants noted their interest in participating in future training to increase research capacity level, as documented in earlier studies [8,9]. In addition, the findings showed that in cases of having a high level of barriers, the opportunity to research among respondents decreased. In this study, identified barriers were consistent with others, including English language barriers [37], lack of research skills [10], lack of research knowledge [4,8], workload [8,10,30], time constraints [10,13,17,20,29], lack of supervision [4], lack of support of a peer group [4], and lack of funding [4,8]. Focusing on specific identified barriers, both personal and organizational barriers, was a priority to improve the practice of the research capacity [6,9,37]. The proportion of respondents having research training (45%) was similar to that reported by a related study (42%) [4]. The study also confirmed the importance of research training, since it was only a predictor of future research among nurses and health professionals. Similar to the findings of Glynn et al., research contributions can affect research activities and capacity, but such opportunities remain contingent on funding, protected time, and training [4]. When the results were combined, they provided meaningful information for planning change and tracking the progress of RCB interventions.

### 4.2. Phase II

#### APTP-RCB Intervention

Developing research capabilities is a broad concept covering planning, development, implementation, evaluation, and sustainability of complex situations [25]. The need assessment of research capacity, barriers, and a predictor of research engagement is baseline data in the planning process and a tool for evaluating research capacity-building intervention [16,37]. Much of the literature on RCB frameworks is available and revealed practical interventions in various places [20,37]. The APTP-RCB intervention embeds training, funding, academic-practice research team, mentoring, and protected time, which are the critical enablers of success in research and RCB outcomes found in the published literature [13,20,24,38]. After engaging in the APTP-RCB intervention, the findings showed that participants perceived competency (PRC) at M2 and M3 higher than those at M1 and M2, respectively. This success may have partly resulted from a nondegree training intervention designed for appropriate contents and focused on discovered competency weak points. However, without a control group and randomization tending to increase internal validity, other factors may have to some extent affected the intervention’s effectiveness (e.g., maturation bias, history bias) and need to be recognized. Though one-group pretest-posttest has the advantage of telling us how much participants achieved during the participation and at the end of intervention, and has often been used in public health, it does have its limitations on confirming a causal association between an intervention and an outcome. Hence, future studies using quasi-experimental design should consider using advanced quasi-experimental designs or randomized controlled trials for higher internal validity [39].

The outcomes of our intervention regarding completing 24 research proposals that developed research questions from participants’ duties and had ethics approval corresponding to critical indicators of developing appropriate skills to highlight research usefulness to practice, contributing research involvement actively, and resulting in a series of research-related outcomes [24,38]. Most participants’ studies were small research projects similar to the findings of Birden’s study that small-scale research projects are more conducive to primary health care research than randomized controlled trials or large-scale observational studies [40]. Thirteen quasi-experimental designs of participants’ projects had impacts improving health behavior/outcomes of their sample engaging in the intervention (e.g., decreasing drug use of patients receiving counseling at methadone clinic, improving maternal breastfeeding behaviors of women experiencing first cesarean section engaged in a program of promoting maternal breastfeeding, decreasing health risk behaviors of patients’ diet and physical activity at a clinic participating in empowerment programs, significantly improving mean quality of nursing documentation after using developed nursing documentation [41], and increasing self-care behaviors of 40 type-2 diabetes patients engaged in a specially designed self-care program [42]).

Research outcomes also depended on participants’ characteristics [20], enthusiasm, and tenacity [5,43]. As participants working in health facilities, completing eight-month APTP-RCB intervention reflected their positive effort to reduce barriers for research engagement rather than working in their comfort zone. Participants’ research involvement and outcomes not only added value to their health practices, professionalism, and organization, but also to the health of patients and society. Their contribution had immediate results for the sake of strengthening health systems. Though several teams had not completed their projects at 32 weeks post-intervention, half of the participants graduated with a master’s degree, and 87.5% had working experience of 20 years or more. These would potentially support participants’ ability to complete a research project with mentors in their team. Previous findings of team-based approach evaluation showed that teams continued their projects beyond the intervention period [13,24].

Our eight-month intervention affected few research disseminations and did not directly target changing policy and practice as suggested by a recent narrative review [15]. Balandya et al. reported on the effectiveness of the first 2 years of a 5-year project where all 12 participants joined in training workshops and proceeded with mentored research awards. Three manuscripts were published in peer reviewed journals [44]. Holden et al. recommended that a two-year course is possibly realistic to bring desirable research outcomes [13]. Feng et al. reported on an 18-month follow-up to post-training initiatives where 11 of 12 participants generated manuscripts published by peer-review international journals [45]. Further strategies targeted at linking research policy and practice may increase the success of RCB aimed at enhancing EBP and emphasizing that evidence must come at least in part from research conducted within the given setting [4]. As such, other possible evaluating indicators of the program such as peer-reviewed publication and embracing evidence to practice and policy need to be studied [20]. In addition, randomized controlled trials would be desirable to know what works and would undoubtedly impact policy [28]. This will increase the sound base of high quality research evidence dissemination to plan services, especially in communities and primary health practices [5,10].

Though some limitations of the one-group pretest-posttest design existed, the outputs of enhancing research competencies, completing writing a research proposal, continuing academic-practice partnership, and quality of research projects approved by ethics committees would be a part of a tangible assessment indicating the effectiveness of this innovative intervention. These successes came from an academic-practice research team and mentoring strategies with milestones to be reached via meetings between mentors and mentees. In addition, tailored training, program funding, and organizations’ supporting time were other successful strategies of our innovative RCB program. Similarly to the notion previously reported, we found that RCB is likely to be successful at any level depending on funding [20,37], mentoring [37], and support opportunities [24].

## 5. Conclusions

According to the purpose of this study, the findings revealed poor research engagement among nurses and health professionals providing communities and primary healthcare services. The significant associations between research experiences, PRC, PRB, and research implementation could serve as useful targets for initiatives aimed at increasing nurses and health professionals’ participation in research development. Factors reducing research barriers were designed as crucial strategies of APTP-RCB intervention. As such, a small grant, organizations’ supporting free time, academic-practice research team, and academic mentors were arranged to decrease barriers. Additionally, both participants of Phases I and II had PRC at a moderate level. The weaknesses in their research capacity in Phase I (e.g., writing a manuscript, developing an instrument, selecting appropriate statistics, designing a project, and interpreting findings) and before engaging in Phase II (e.g., conceptualizing a research framework) were tailored in the nondegree training contents.

The APTP-RCB program, established based on vital gaps and composed of research training, a small grant, academic-practice research team, protecting time, and mentoring effectively helped to eliminate participants’ weak points regarding both competency and barriers. As 23 projects were approved by the institutional review board reflecting the quality of research projects, it indicated that participants could formulate research questions and assess and use more empirical evidence in their research projects. However, only 12.5% had presented research findings in academic conferences and 25% had prepared a manuscript for a regional academic conference. It partly reflects that though time constraints are being overcome, they remain in these limited-resource settings. A recommendation is that it would likely be beneficial to extend the duration of the RCB program’s monitoring and evaluation in health facilities with limited health resources. Additional qualitative research is also required to gain a better understanding of research management strategies.

Based on the results obtained from this study, the disconnect between healthcare evidence and practice is detrimental to health quality. Research and RCB will continue to move forward, and EBP’s will increase in an effort to value overall health outcomes. This study further highlighted the critical issue of nurses and health professionals in rural areas being under-engaged in research development. Many nurses and healthcare professionals have not yet accessed research training. A feasible RCB intervention needs to be further developed according to a needs assessment and feasibility study. An innovative APTP-RCB program can help nurses and other health professionals improve their research competencies. The RCB intervention showed more fruitful outcomes such as increasing research competency, proposals, and implementation, and developing academic-practice partnerships.

The RCB was a complex intervention critiqued to make greater efforts to evaluate the program regarding generalizability versus context framework, methods, tools, and instruments [16,24]. Moreover, RCBs should be tailored according to identified barriers in a specific context. Given appropriate organization and individual environments, nurses and healthcare professionals are ready to be developed. Additionally, compatibility between the organization and career development should be emphasized and incorporated into individual and institutional appraisal processes. Further studies should pay more attention to all health professionals to benefit from reducing barriers, generating health research, and promoting initiative changes in practice and policy for desired health outcomes. A long term training course might prove effective in terms of research completeness, the ability to publish in peer-reviewed journals, and impacts on health policy and practices as well as the health outputs and outcomes of users.

To the best of our knowledge, this is the first ever study to explore the significance of research training and support of RCB in Nakhon Si Thammarat Province. The study organizers successfully coordinated with leaders of the district hospital, the provincial hospital, the District Public Health Office, and the Provincial Public Health Office to ensure adequate time or research practice among staff nurses. However, some limitations of this study are that the needs assessment did not include all health professionals working in the district hospital, which may not reflect the actual picture/situation of this research. A larger sample size is recommended, especially to include all health professionals at provincial, regional, or national levels. Evaluation of the APTP-RCB program focused on the outputs similar to Sombie et al.’s study [46] linkage between health research, policy and practices should be carried out further. A single pre- and posttest study group was used to compare the participants’ research competency and outputs with limitations. Though commonly used in the field of social work, public health, and education, the difference between pre-and posttest may have been affected by other confounding factors such as history, maturation, and testing effects [47]. Additionally, the eight-month APTP-RCB program had a dropout rate of 46.7%, similar to the study by Melender et al. [48] who reported a dropout rate of 42.2% after participating in their six month intervention. This may be due to time-consuming and routine workload as frequently encountered in limited health resources settings.

Despite having limitations, the APTP-RCB program could identify crucial factors that increased participants’ ability to conduct research within their practices that benefit patients. The findings reaffirm the importance of research in developing nurses’ and health professionals’ ability to conduct high-quality research. The relationships between research experience and involvement in research can be useful for identifying groups within the community and primary healthcare facilities that should be targeted for enhancing research implementation. Interest in future research training was critical, and ongoing RCB is still required to motivate nurses and healthcare professionals to develop, maintain, and apply their research skills.

## Figures and Tables

**Table 1:** Respondents’ personal characteristics and research experiences (*n* = 149).

Characteristic (Mean ± SD)	Category (Subcategories)	*n* (%)
**Personal** **factors**		
Age (years) (37.6 ± 9.8)	<40	83 (55.7%)
	≥40	66 (44.3%)
Gender	Female	127 (85.2%)
	Male	22 (14.8%)
Education	Bachelor degree or less	137 (91.9%)
	Master degree	12 (8.1%)
Marital status	Married/separated/widowed/divorced	94 (63.1%)
	Single	55 (36.9%)
Monthly income	>30,000 THB	83 (55.7%)
	≤30,000 THB	66 (44.3%)
Working years (14.9 ± 10.1)	<19	92 (61.7%)
	≥19	57 (38.3%)
Health care setting	District hospital	110 (73.8%)
	Subdistrict Health Promoting Hospital	39 (26.2%)
Working position	Professional nurse	96 (64.4%)
	Other health professional	53 (35.6%)
	(public health officer)	33 (22.2%)
	(public health technical officer)	8 (5.4%)
	(medical technologist)	5 (3.4%)
	(medical science technician)	3 (2.0%)
	(physiotherapist)	2 (1.3%)
	(occupational therapist)	2 (1.3%)
**Research Experience**		
Training in research	Never	82 (55.0%)
	Had been trained	67 (45.0%)
Attending research conference	Had attended	101 (67.8%)
	Never	48 (32.2%)
Using research findings	Had used	88 (59.1%)
	Never	61 (40.9%)
Research implementation	Never	99 (66.4%)
	Had implemented	50 (33.6%)
	(a research project being undertaken)	10 (6.7%)
Interest in participating in future research training	Yes	103 (69.1%)
	No	46 (30.9%)

**Table 2:** Perceived Research Capacity (PRC) and Perceived Research Barriers (PRB).

The Domain and Selected Items of PRC and PRB	Total Score	Mean	SD	Level
Total Perceived Research Capacity (PRC)	100	58.2	12.8	moderate
Literature reviews and research framework (RC2-LrRf)	100	60.2	13.7	moderate
Research problems, purpose, and hypotheses (RC1-RpPH)	100	59.7	13.0	moderate
Data collection, analysis, and interpretation (RC4-DcAI)	100	59.0	13.0	moderate
Research method (RC3-Rm)	100	56.7	12.9	moderate
Finding’s discussion and dissemination (RC5-FdD)	100	55.9	14.3	moderate
Total Perceived Research Barriers Subscale (PRB)	100	64.2	11.2	moderate
Organizational barriers (RB2-Ob)	100	66.6	12.8	moderate
Personal barriers (RB1-Pb)	100	61.8	12.0	moderate
5-items of PRC holding the lowest mean				
Writing a research manuscript	5	2.6	0.8	moderate
Preparing a research instrument	5	2.6	0.7	moderate
Selecting appropriate statistics	5	2.7	0.8	moderate
Designing a research project	5	2.7	0.7	moderate
Interpreting findings	5	2.8	0.8	moderate
Top five barriers of RB1-Pb				
Reading English research literature	5	3.9	1.0	highest
Lack of research experience	5	3.6	1.0	highest
Abundance of research procedures	5	3.5	0.9	highest
Little knowledge of research methods	5	3.5	0.9	highest
Identifying research problems	5	3.3	0.8	moderate
Top five barriers of RB2-Ob				
Workload constraints	5	3.7	1.0	highest
Time burden	5	3.6	0.8	highest
No research team	5	3.6	0.9	highest
Lack of research mentors	5	3.6	0.9	highest
Research funding	5	3.5	1.0	highest

**Table 3:** Associations between personal factors, research experiences, and research implementation (*n* = 149).

Characteristic (Mean ± SD)	Category (Subcategories)	Never Implemented Research *n* (%)	Had Implemented Research *n* (%)	χ^2^ (*p*-Value)
**Personal factors**				
Age (years) (37.6 ± 9.8)	<40	58 (58.6%)	25 (50.0%)	0.993 (0.319)
	≥40	41 (41.4%)	43 (50.0%)	
Sex	Male	15 (15.2%)	7 (14.0%)	0.035 (0.852)
	Female	84 (84.8%)	43 (86.0%)	
Education	Bachelor degree and lower	92 (92.9%)	45 (90.0%)	(0.538) ^a^
	Master degree	7 (7.1%)	5 (10.0%)	
Marital status	Single	37 (37.4%)	18 (36.0%)	0.027 (0.870)
	Married/Separated/Widowed	62 (62.6%)	32 (64.0%)	
Monthly income	30,000 THB	47 (47.5%)	19 (38.0%)	1.209 (0.272)
	>30,000 THB	52 (52.5%)	31 (62.0%)	
Working years (14.9 ± 10.1)	<19	66 (66.7%)	26 (52.0%)	3.025 (0.082)
	≥19	33 (33.3%)	24 (48.0%)	
Health care setting	Subdistrict Health Promoting Hospital	24 (24.2%)	15 (30.0%)	0.570 (0.450)
	District hospital	75 (75.8%)	35 (70.0%)	
Working position	Professional Nurse	64 (64.6%)	32 (64.0%)	0.006 (0.938)
	Other health professional	35 (35.4%)	18 (36.0%)	
**Research experience**				
Training in research	Never	71 (71.7%)	11 (22.0%)	33.183 (<0.001) ***
	Had been trained	28 (28.3%)	39 (78.0%)	
Attending research conference	Never	43 (43.4%)	5 (10.0%)	17.006 (<0.001) ***
	Had attended	56 (56.6%)	45 (90.0%)	
Using research findings	Never	49 (49.5%)	12 (24.0%)	8.931 (0.003) **
	Had used	50 (50.5%)	38 (76.0%)	
Interest in participating in future research trainings	No	36 (36.4%)	10 (20.0%)	4.168 (0.041) *
	Yes	63 (63.6%)	40 (80.0%)	

^a^ Fisher’s exact test. * *p* < 0.05, ** *p* < 0.01, *** *p* < 0.001.

**Table 4:** Association between PRC and research implementation.

Domain of Perceived Research Capacity	Research Implementation	Mean Rank	Sum of Ranks	*Z*-Test	*p*-Value
Research problems, purpose, and hypotheses (RC1-RpPH)	Never	70.8	7013.0	−1.676	0.047 *
	Implemented	83.2	4162.0		
Literature reviews and research framework (RC2-LrRf)	Never	70.6	6990.0	−1.762	0.039 *
	Implemented	83.7	4185.0		
Research method (RC3-Rm)	Never	72.3	7158.5	−1.075	0.141
	Implemented	80.3	4016.5		
Data collection, analysis, and interpretation (RC4-DcAI)	Never	73.2	7243.0	−0.738	0.230
	Implemented	78.6	3932.0		
Finding’s discussion and dissemination (RC5-FdD)	Never	73.0	7230.0	−0.790	0.215
	Implemented	79.0	3945.0		
Total Perceived Research Capacity (PRC)	Never	72.1	7137.0	−1.158	0.124
	Implemented	80.1	4038.0		

* *p* < 0.05.

**Table 5:** Association between PRB and research implementation.

Domain of Perceived Research Barriers Subscale	Never		Had		*t*-Test	*p*-Value
	Mean	SD	Mean	SD		
Total Perceived Research Barriers Subscale (PRB)	65.6	10.5	61.4	12.1	2.125	0.018 *
Personal barriers (RB1-Pb)	63.4	10.4	58.6	14.2	2.297	0.012 *
Organizational barriers (RB2-Ob)	67.9	13.0	64.1	12.2	1.751	0.042 *

* *p* < 0.05.

**Table 6:** Coefficient of variables to predict research implementation.

Variable	Coefficient	SE	*p*-Value	Odds Ratio
Training in research	1.761	0.464	<0.001 ***	5.8
Attending research conference	0.916	0.596	0.125	2.5
Using research findings	0.458	0.457	0.316	1.6
Research problems, purpose, and hypotheses (RC1-RpPH)	−0.039	0.070	0.579	1.0
Organizational barriers (RB2-Ob)	0.013	0.035	0.721	1.0
Personal barriers (RB1-Pb)	−0.036	0.037	0.346	1.0
Interest in participating in future research trainings	0.015	0.482	0.744	1.1

Constant = −1.534, *** *p* < 0.001.

**Table 7:** Personal characteristics of respondents participating in the APTP-RCB interventions (*n* = 24).

Characteristics (Mean± SD)	Categories	*n* (%)
Age (years) (47.8 ± 4.3)	≥40	22 (91.7%)
	<40	2 (8.3%)
Sex	Female	24 (100.0%)
	Male	0 (0.0%)
Education	Bachelor’s degree	11 (45.8%)
	Master’s degree	13 (54.2%)
Marital status	Married	19 (79.2%)
	Single	4 (16.6%)
	Separated/widowed/divorced	1 (4.2%)
Monthly income (THB)	>50,000	10 (41.7%)
	40,001–50,000	5 (20.8%)
	30,001–40,000	7 (29.2%)
	20,001–30,000	2 (8.3%)
Working years (25.6 ± 4.9)	≥20	21 (87.5%)
	<20	3 (12.5%)
Health care setting	Provincial hospital	14 (58.3%)
	District hospitals	6 (25.0%)
	Nursing College	3 (12.5%)
	Sub-district Health Promoting Hospitals	1 (4.2%)
Working position	Professional Nurse	18 (75.0%)
	Public health officers	2 (8.3%)
	Clinical psychologist	2 (8.3%)
	Pharmacist	1 (4.2%)
	Medical technologists	1 (4.2%)

**Table 8:** Perceived Research Capacity (PRC) (*n* = 24).

Domain and Selected Items of PRC	Total Score	Mean	SD	Level
Total Perceived Research Capacity (PRC)	100	50.4	15.7	moderate
Data collection, analysis, and interpretation (RC4-DcAI)	100	56.9	16.4	moderate
Research problems, purpose, and hypotheses (RC1-RpPH)	100	52.5	18.3	moderate
Literature reviews and research framework (RC2-LrRf)	100	51.0	17.5	moderate
Research method (RC3-Rm)	100	51.6	17.3	moderate
Findings discussion and dissemination (RC5-FdD)	100	49.8	16.1	low
5 items holding the lowest mean				
Conceptualizing a research framework	5	2.3	0.9	low
Selecting appropriate statistics	5	2.3	0.9	low
Writing a research manuscript	5	2.3	0.9	low
Designing a research project	5	2.4	0.9	low
Preparing a research instrument	5	2.4	0.9	low

**Table 9:** Mean scores of participants’ PRC before, after 16, and after 32 weeks of the program (*n* = 24).

Domain of Perceived Research Capacity	Time of Measurement	Mean	SD		*p*-Value	
				M1-M2-M3 ^a^	M1-M2 ^b^	M2-M3 ^b^
Research problems, purpose, and hypotheses (RC1-RpPH)	M1	50.4	15.7	<0.001 ***		
	M2	72.3	10.5		<0.001 ***	
	M3	76.4	9.3			0.112
Literature reviews and research framework (RC2-LrRf)	M1	51.0	17.5	<0.001 ***		
	M2	75.3	9.3		<0.001 ***	
	M3	77.5	9.0			0.295
Research method (RC3-Rm)	M1	51.6	17.3	<0.001 ***		
	M2	72.6	11.0		<0.001 ***	
	M3	77.2	10.0			0.008 **
Data collection, analysis, and interpretation (RC4-DcAI)	M1	56.9	16.4	<0.001 ***		
	M2	74.2	12.0		<0.001 ***	
	M3	77.1	11.1			0.169
Findings discussion and dissemination (RC5-FdD)	M1	49.8	16.1	<0.001 ***		
	M2	68.7	11.1		<0.001 ***	
	M3	74.0	10.5			0.020 *
Total Perceived Research Capacity (PRC)	M1	50.4	15.7	<0.001 ***		
	M2	72.3	10.5		<0.001 ***	
	M3	76.4	9.3			0.014 *

^a^, Friedman test; ^b^, Wilcoxon sign rank test; * *p* < 0.05; ** *p* < 0.01; *** *p* < 0.001.

**Table 10:** Outputs of APTP-RCB intervention at 32 weeks (*n* = 24 projects).

Characteristic	Categories	*n* (%)
Ethics approval	Approved	23 (95.8%)
	Being revised	1 (4.2%)
Received financial support	Yes	22 (91.7%)
	No	2 (8.3%)
Research design	Cross sectional study	11 (45.8%)
	Quasi-experimental	13 (54.2%)
Targeted sample	Health services recipients (e.g., patients received counseling at methadone clinic, women experiencing first cesarean section, patients of diet and physical activity clinic)	16 (66.7%)
	Community residents/leaders	3 (12.5%)
	Public health professionals	2 (8.3%)
	Donor blood sample	1 (4.2%)
	Medical records	2 (8.3%)
Research dissemination	International academic conference	2 (8.3%)
	Thai academic conference	1 (4.2%)
	Manuscripts being prepared for regional academic conference	6 (25.0%)
	In progress	15 (62.5%)

## Data Availability

The data presented in this study are available on request from the corresponding author. The data are not publicly available because this issue was not considered within the informed consent signed by the participants of the study.
